# Metformin in breast cancer - an evolving mystery

**DOI:** 10.1186/s13058-015-0598-8

**Published:** 2015-06-26

**Authors:** Laura Camacho, Atreyi Dasgupta, Sao Jiralerspong

**Affiliations:** Lester and Sue Smith Breast Center, Department of Medicine, Baylor College of Medicine, Houston, TX 77030 USA; Department of Medicine, Baylor College of Medicine, Houston, TX 77030 USA

## Abstract

Metformin, a diabetes drug with well-established side effect and safety profiles, has been widely studied for its anti-tumor activities in a number of cancers, including breast cancer. But its mechanism of action in the clinical arena remains elusive. In a window of opportunity trial of metformin in non-diabetic breast cancer patients, Dowling and colleagues examined both the direct actions of the drug on cancer cells (as mediated by AMP kinase), as well as its indirect actions (as mediated by circulating insulin). The data suggest that short-term administration of metformin in this setting has anti-tumor effects significantly involving the indirect, insulin-dependent pathway. The role of the direct pathway remains to be determined. This study represents an important step forward in establishing one of several possible mechanisms for metformin, information that will be useful in determining candidate biomarkers to evaluate in large clinical trials of metformin, such as the ongoing NCIC CTG MA.32 trial of adjuvant metformin. The potential significance of these data for metformin in the treatment of breast cancer is discussed here.

Metformin has been studied in breast cancer, but its mechanism of action in the clinical arena remains unclear. Several trials have attempted to address this knowledge gap (Table 1) [1–10]. In a recent issue of *Breast Cancer Research*, Dowling and colleagues [10] present their mechanistic studies from a previously reported single arm, neoadjuvant, window of opportunity trial of metformin in non-diabetic breast cancer patients. Thirty-nine operable breast cancer patients were given metformin 500 mg three times daily for a median of 18 days (range 13 to 40). Their results demonstrate that short-term administration of metformin in this setting has anti-cancer properties significantly involving indirect actions of metformin.

**Table 1:** Metformin trials in non-diabetic breast cancer patients

Study/reference	Number of women	Study population	Setting	Design	Metformin dosing	Weight	Serum glucose	Serum insulin	HOMA	Proliferation (Ki-67)	Apoptosis	Insulin-dependent actions of metformin (indirect)	Insulin-independent actions of metformin (direct)
Goodwin *et al.* 2008 [1]	22	Early BC patients; insulin >45 pmol/L	Adjuvant	Single arm	500 mg tid × 6 mos	↓	**○**	↓	↓				
Hadad *et al.*, 2011 [2], Hadad *et al*. 2015 [3]	8 + 47	Operable invasive BC	Neoadjuvant window	Metf versus ctrl (no metf)	500 mg qd × 1 wk, then 1000 mg bid × 1 wk			○ in metf, ↑ in ctrl		↓ in metf, ○ in ctrl	↓Cleaved Caspase-3	NS ↓ IR; ↓ pAkt	↑p-AMPK
Bonanni *et al.* 2012 [4], Cazzaniga *et al.* 2013 [5], DeCensi *et al.* 2014 [6]	200	Operable BC	Neoadjuvant window	Metf versus ctrl (placebo)	850 mg bid × 4 wks		↓ in BMI > 27	NS ↓ in BMI > 27		○ metf versus ctrl; NS ↓ in HOMA > 2.8 and NS ↑ in HOMA < 2.8	TUNEL ↑ in both metf and ctrl	↓IGFBP-1	
Kalinsky *et al.* 2014 [7]	35	Ov/Ob invasive BC or DCIS versus matched untreated historical ctrl	Neoadjuvant window	Single arm	500 mg am and 1000 mg pm, 2–4 wks (avg = 22 days)	↓	○	NS ↓	NS ↓	○		○ IGFBP-3	
Goodwin *et al.* 2015 [8]	3,649	Treated early BC	Adjuvant	Metf versus ctrl (placebo)	850 mg bid × 5 yrs (results reported after 6 mos)	↓	↓	↓	↓				
Niraula *et al*. 2012 [9], Dowling *et al.* 2015 [10]	39	Operable BC	Neoadjuvant window	Single arm	500 mg tid 13–40 days (avg = 18)	↓	↓	NS ↓	↓	Largest ↓ in patients with largest ↓ in insulin, IR, and pAkt	TUNEL ↑	↓ IR, pAkt, and pERK1/2	↓ pAMPK and pACC; all tumors expressed OCT1

Down arrows indicate decrease; circles indicate no change; up arrows inidcate increase. ACC, acetyl-CoA carboxylase; AMPK, AMP-activated protein kinase; avg, average; BC, breast cancer; bid, twice a day; BMI, body mass index; ctrl, control; DCIS, ductal carcinoma *in situ*; ERK, extracellular signal-regulated protein kinase; HOMA, homeostatic model assessment; IGFBP, insulin-like growth factor-binding protein; IR, insulin receptor; metf, metformin; mos, months; NS, non-significant; Ob, obese; OCT, organic cation transporter; Ov, overweight; qd, once a day; tid, three times a day; TUNEL, terminal deoxynucleotidyl transferase (TdT) dUTP nick-end labeling; wks, weeks; yrs, years

By binding to its receptor, insulin has been shown to have mitogenic and anti-apoptotic effects in several cancers, including breast cancer, and circulating insulin is associated with increased cancer risk and prognosis [11]. In their previous publication on the same trial [9], the authors reported significant decreases in weight, body mass index (BMI), glucose, homeostatic model assessment (HOMA), and tumor cell proliferation by Ki-67, as well as an increase in apoptosis by TUNEL (terminal deoxynucleotidyl transferase (TdT) dUTP nick-end labeling) after metformin treatment. In the current study, they point to the insulin-dependent effects of metformin based on the decrease in insulin receptor (IR) expression in tumors together with reductions in Akt and ERK1/2 phosphorylation (key downstream effectors of phosphatidylinositol 4,5-bisphosphate 3-kinase (PI3K)/Akt and Ras-mitogen-activated protein kinase (MAPK) signaling pathways, respectively). Furthermore, in an important analysis, when patients were assessed individually, the largest decreases in serum insulin, tumor IR, and p-Akt (via a summary score of all three variables) correlated with the largest decreases in tumor cell proliferation. However, overall changes in Akt and ERK1/2 phosphorylation did not correlate with reductions in circulating insulin levels, suggesting that these changes were also mediated by additional (unexplored) insulin-independent pathways.

The decreases in Akt and ERK1/2 phosphorylation are in agreement with previous *in vitro* and *in vivo* breast cancer studies [12, 13]. Likewise, the decreases in IR expression and p-Akt are consistent with a two arm window of opportunity trial conducted by Bonanni and colleagues [4], where non-diabetic women with breast cancer were randomized to metformin or no drug. In that study, a non-significant decrease in IR and a significant decrease in p-Akt were found in the metformin group, while non-significant increases were found in the control group. In future studies, it would be interesting to analyze activated (phosphorylated) IR staining in tumors to further define the role of this signaling axis.

Metformin can also exert its anti-tumor activity through insulin-independent direct actions on cancer cells. The most studied pathway involves the serine/threonine kinase AMP-activated protein kinase (AMPK), a metabolic master switch which is activated in low energy states. Upon activation, AMPK increases cellular energy levels by inhibiting energy-consuming anabolic pathways and stimulating energy-producing catabolic pathways. The role of AMPK in tumorigenesis is the subject of ongoing investigation. Metformin has been shown to activate AMPK in both cancer and non-cancer settings. This includes a window of opportunity trial by Hadad and colleagues [3], where treated patients showed significant upregulation of p-AMPK compared with the control group. In the current study, contrary to expectation, the authors found a decrease in the activation of AMPK and one of its targets (acetyl-CoA carboxylase(ACC)) upon treatment with metformin, as well as a high baseline level of AMPK. A similar result was obtained in a window of opportunity study in endometrial cancer [14]. The explanation for this paradoxical result is unclear. However, it is known that metformin may have both AMPK-dependent and AMPK-independent anti-tumor effects in different contexts [15]. AMPK-independent effects were not examined in the current study and should be the subject of future investigations of the direct effects of metformin in breast cancer.

The current study has a number of strengths. Metformin was administered as close as possible to the time of tumor tissue acquisition, which may be critical for accurate measurement of biomarkers. The analysis in individual patients to correlate several biomarkers (serum insulin, and tumor IR and p-Akt, with tumor Ki-67) is a compelling one. Limitations include small sample size and lack of no treatment controls, so that the results will need to be validated in larger trials. The study suggests some additional analyses to keep in mind for future trials. The pharmacology of metformin in the cancer setting is largely unexplored. It would be interesting to see whether dividing the wide range of metformin treatment time (13 to 40 days) into groups of shorter and longer duration or into high versus low OCT1 expression levels would reveal differences in biomarkers according to these parameters.

In summary, in combination with the results from the trials listed in Table 1, the results of Dowling *et al*. strongly suggest that metformin exerts a significant part of its anti-tumor effects in breast cancer via the indirect pathway by lowering serum insulin, inhibiting downstream signaling via PI3K/Akt and Ras-MAPK pathways, and leading to decreases in cell proliferation. This information has important implications for the conduct and analysis of ongoing and future clinical trials of metformin. A number of trials are currently underway evaluating metformin in breast cancer (clinicaltrials.gov). The largest and most advanced is the NCIC CTG MA.32, an ongoing adjuvant trial of 3,649 women with early stage breast cancer examining the effects of metformin versus placebo on survival and other outcomes. An initial report has been published showing that metformin significantly improved metabolic parameters such as weight, insulin, glucose, leptin, and C-reactive protein (CRP) at 6 months, regardless of initial weight or degree of insulin resistance [8]. The results of the current study suggest that serum insulin and tumor IR, p-Akt, and Ki-67 should be evaluated as potential biomarkers of metformin tumor sensitivity in this and other metformin trials in breast cancer. The role of the direct effects of metformin on breast cancer cells, whether mediated via AMPK or otherwise, remains an open question to be explored in future pre-clinical and clinical studies. These and other studies will undoubtedly contribute to the evolving mystery of this fascinating drug.

## Abbreviations

AMPK: AMP-activated protein kinase
IR: insulin receptor
MAPK: mitogen-activated protein kinase
PI3K: phosphatidylinositol 4,5-bisphosphate 3-kinase

## References

[1] Goodwin PJ, Pritchard KI, Ennis M, Clemons M, Graham M, Fantus IG (2008). Insulin-lowering effects of metformin in women with early breast cancer. Clin Breast Cancer.

[2] Hadad S, Iwamoto T, Jordan L, Purdie C, Bray S, Baker L (2011). Evidence for biological effects of metformin in operable breast cancer: a pre-operative, window-of-opportunity, randomized trial. Breast Cancer Res Treat.

[3] Hadad SM, Coates P, Jordan LB, Dowling RJ, Chang MC, Done SJ (2015). Evidence for biological effects of metformin in operable breast cancer: biomarker analysis in a pre-operative window of opportunity randomized trial. Breast Cancer Res Treat.

[4] Bonanni B, Puntoni M, Cazzaniga M, Pruneri G, Serrano D, Guerrieri-Gonzaga A (2012). Dual effect of metformin on breast cancer proliferation in a randomized presurgical trial. J Clin Oncol.

[5] Cazzaniga M, DeCensi A, Pruneri G, Puntoni M, Bottiglieri L, Varricchio C (2013). The effect of metformin on apoptosis in a breast cancer presurgical trial. Br J Cancer.

[6] DeCensi A, Puntoni M, Gandini S, Guerrieri-Gonzaga A, Johansson HA, Cazzaniga M (2014). Differential effects of metformin on breast cancer proliferation according to markers of insulin resistance and tumor subtype in a randomized presurgical trial. Breast Cancer Res Treat.

[7] Kalinsky K, Crew KD, Refice S, Xiao T, Wang A, Feldman SM (2014). Presurgical trial of metformin in overweight and obese patients with newly diagnosed breast cancer. Cancer Invest.

[8] Goodwin PJ, Parulekar WR, Gelmon KA, Shepherd LE, Ligibel JA, Hershman DL (2015). Effect of metformin vs placebo on and metabolic factors in NCIC CTG MA.32. J Natl Cancer Inst.

[9] Niraula S, Dowling RJ, Ennis M, Chang MC, Done SJ, Hood N (2012). Metformin in early breast cancer: a prospective window of opportunity neoadjuvant study. Breast Cancer Res Treat.

[10] Dowling RJ, Niraula S, Chang MC, Done SJ, Ennis M, McCready DR (2015). Changes in insulin receptor signaling underlie neoadjuvant metformin administration in breast cancer: a prospective window of opportunity neoadjuvant study. Breast Cancer Res.

[11] Pollak M (2008). Insulin and insulin-like growth factor signalling in neoplasia. Nat Rev Cancer.

[12] Zhu P, Davis M, Blackwelder AJ, Bachman N, Liu B, Edgerton S (2014). Metformin selectively targets tumor-initiating cells in ErbB2-overexpressing breast cancer models. Cancer Prev Res (Phila).

[13] Alimova IN, Liu B, Fan Z, Edgerton SM, Dillon T, Lind SE, Thor AD (2009). Metformin inhibits breast cancer cell growth, colony formation and induces cell cycle arrest in vitro. Cell Cycle.

[14] Schuler KM, Rambally BS, DiFurio MJ, Sampey BP, Gehrig PA, Makowski L, Bae-Jump VL (2015). Antiproliferative and metabolic effects of metformin in a preoperative window clinical trial for endometrial cancer. Cancer Med.

[15] Hardie DG, Alessi DR (2013). LKB1 and AMPK and the cancer-metabolism link - ten years after. BMC Biol.

